# Effect of nicorandil administration on cardiac burden and cardio-ankle vascular index after coronary intervention

**DOI:** 10.1007/s00380-020-01650-9

**Published:** 2020-06-22

**Authors:** Shuji Sato, Mao Takahashi, Hiroshi Mikamo, Masayo Kawazoe, Takuo Iizuka, Kazuhiro Shimizu, Mahito Noro, Kohji Shirai

**Affiliations:** 1 Department of Cardiology, Toho University Medical Center Sakura Hospital, 564-1 Shimoshizu, Sakura, Chiba 285-8741 Japan; 2 Mihama Hospital, Chiba, Japan

**Keywords:** Nicorandil, Cardiac burden, Cardio-ankle vascular index, Percutaneous coronary intervention, Myocardial injury, Angina

## Abstract

Myocardial injury is a problem associated with percutaneous coronary intervention (PCI). This study aimed to clarify the role of nicorandil administration in preventing myocardial injury. This study included patients with stable angina who underwent PCI from November 2013 to June 2016. Of 58 consecutive patients, the first 20 patients received only saline infusion after PCI (control group); the other 38 patients received a continuous intravenous infusion of nicorandil and saline after PCI (nicorandil group). Troponin I and brain natriuretic peptide (BNP) levels were measured. Vascular parameters, such as blood pressure (BP), cardiac output, cardio-ankle vascular index (CAVI), and estimated systemic vascular resistance (eSVR), were measured. Troponin I of both groups increased 12 h after PCI. Changes in BNP levels between immediately after PCI and 12 h after PCI were significantly higher in the control than in the nicorandil group (10.8 ± 44.2 vs. − 2.6 ± 14.6 pg/ml, *p* = 0.04). In the nicorandil group, BP, eSVR, and CAVI decreased significantly at 12 h after PCI compared with those immediately after PCI (*p* < 0.0001), whereas no change was observed in the control group. In a single linear analysis, the change in BP (*r* = 0.36, *p* < 0.01) and nicorandil administration (*r* = − 0.47, *p* < 0.001) was significantly correlated with the change in CAVI, multiple regression analysis revealed that the changes in CO and eSVR were significant contributing factors for the changes in CAVI. PCI could result in myocardial injury and/or cardiac burden in patients with stable angina. Nicorandil administration after PCI may be effective in relieving the burden by decreasing arterial stiffness (CAVI).

## Introduction

Percutaneous coronary intervention (PCI) for ischemic heart disease has made remarkable progress since it was first introduced in 1977 [1]. Currently, stent thrombosis and in-stent restenosis, which were major problems in the early stages of PCI, have been greatly reduced by the improvement of therapeutic devices, such as drug-eluting stents, and by additional medical therapy using antiplatelet agents and statins [2–6] In addition, techniques for complex coronary artery lesions, such as bifurcation and chronic total occlusive lesions, have been advanced and standardized [7, 8]. Thus, PCI has undergone development.

However, myocardial injury remains a problem in coronary intervention [9]. When devices, such as a balloon catheter or stent, are inserted into the coronary artery lumen, and balloon dilation or stent expansion is performed, coronary plaques are sometimes crushed, and myocardial ischemia may occur with embolism due to the ruptured plaque fragments. Moreover, vessel injuries, such as coronary artery dissection and hematoma, may also occur. Stent deployment for bifurcation lesion may also result in side branch occlusion, which could be due to plaque shift or carina shift. All these are problems associated with even advanced PCI techniques and often cause procedure-related myocardial injury, which in turn leads to cardiac dysfunction or heart failure. At times, care of post-PCI complications is necessary.

Nicorandil is a nicotinamide ester that dilates peripheral and coronary resistance vessels via its action on ATP-sensitive potassium channels and possesses a nitrate moiety that promotes systemic venous and coronary vasodilation [10, 11]. Nicorandil improves coronary microcirculation and is reported to be effective in patients with stable angina and acute coronary syndrome [12]. Furthermore, nicorandil exerts a vasodilating action on systemic vessels to reduce preload and afterload and to increase cardiac output, and it was reported that nicorandil is also useful as a therapeutic agent for acute heart failure [13, 14]. Because of its pharmacological properties, nicorandil is supposed to be useful for preventing the progression of myocardial injury and relieving cardiac burden after PCI.

As a biomarker for heart failure, brain natriuretic peptide (BNP) is used in daily clinical practice, and its usefulness as an index of heart failure treatment has been established by its relationship to the state and prognosis of heart failure [15]. Moreover, in the treatment of heart failure, evaluating the preload and afterload on the heart in addition to cardiac function assessment is essential. Preload could be objectively evaluated using a Swan-Ganz catheter or echocardiography. The main components of afterload include systolic blood pressure (BP), vessel resistance, and arterial stiffness; blood pressure and vessel resistance could be measured invasively or non-invasively. Although arterial stiffness is also an important factor of afterload and is considered to relate to heart failure onset, a method for evaluating arterial stiffness has not been established [16].

Cardio-ankle vascular index (CAVI) was developed as a new index of arterial stiffness, i.e., from the aortic root to the ankle, independent of BP at the time of measurement [17]. CAVI is clinically used as a surrogate marker for arteriosclerosis because its value increases with organic atherosclerosis development [18]. Furthermore, the value of CAVI is decreased by the administration of some vasodilators. These results suggest that CAVI reflects not only organic arterial stiffness due to arteriosclerosis progression but also functional arterial stiffness due to vascular smooth muscle relaxation or contraction [19–22]. Thus, arterial stiffness, which can be monitored with CAVI, may partly reflect the after-loading of the left ventricle and may be involved in the control of systemic hemodynamics; however, the details are unclear.

Hence, this study aimed to clarify the effect of nicorandil on myocardial injury and cardiac burden after PCI in patients with stable angina and to determine the relationship between cardiac burden and arterial stiffness (CAVI).

## Materials and methods

The subjects in this observational study were patients with stable angina who underwent PCI at Toho University Medical Center Sakura Hospital from November 2013 to June 2016; CAVI measurements were made on 58 consecutive patients before, immediately after, and 12 h after PCI. Among them, the first 20 consecutive patients received only saline infusion after PCI (control group), and the other 38 patients received a continuous intravenous infusion of nicorandil and saline after PCI (nicorandil group). In the nicorandil group, a bolus of 5 mg nicorandil was administered immediately after PCI, which was followed by continuous intravenous administration of nicorandil 2 mg/h or 4 mg/h for 12 h. Saline was continuously administered intravenously (40–80 ml/h) immediately after PCI for 12 h in both groups. Patients with atrial fibrillation, those with a peripheral arterial disease with ankle–brachial index < 0.9, those undergoing dialysis, and those with mechanical circulatory assist devices were excluded.

CAVI measurements were performed in all patients before PCI, immediately after PCI (before nicorandil administration), and 12 h after PCI using VaSela VS 1500 (Fukuda Denshi Co. Ltd, Tokyo, Japan). During CAVI measurement, systolic BP, diastolic BP, mean BP, heart rate, and stroke volume were also measured. Stroke volume was non-invasively measured using an impedance cardiac output meter (Aesculon, Osypka Medical Inc., Berlin, Germany) after 10 min of supine rest. From the measured data, cardiac output (CO) was calculated. In the calculation of estimated systemic vascular resistance (eSVR), a mean right atrial pressure of 8 mmHg was used.

Troponin I, which is a biomarker for myocardial injury, was measured before and 12 h after PCI. BNP, which is a biomarker of cardiac burden, was measured before, immediately after, and 12 h after PCI. Blood samples were collected after resting for ≥ 10 min. PCI was performed in a standard manner. At the discretion of the operator, either the radial artery approach or femoral artery approach was employed, and CAVI values opposite to the puncture side were used in the analysis. None of the patients had puncture sites at both radial and femoral arteries. All patients underwent PCI while receiving their usual oral medications. Drugs that are necessary during PCI were administered at the discretion of the operator. Patients who received catecholamine to maintain hemodynamics were not included.

Informed consent was obtained from all patients, and this study was approved by the ethics committee of Toho University Medical Center Sakura Hospital (S17058) and performed in accordance with the Declaration of Helsinki.

### Statistical analysis

Continuous variables were expressed as mean ± standard deviation. Categorical variables were expressed as percentages. Unpaired *t* test and Wilcoxon test were used for the comparison of normally and non-normally distributed continuous variables, respectively. Chi-square test was used for ratio comparison, and Fisher's exact test was used when the expected value was < 5. Data before and after PCI and after nicorandil administration were compared using analysis of variance, and post hoc Bonferroni test was performed. The association between changes in CAVI (∆CAVI) and various circulation parameters (∆BP, ∆CO, ∆eSVR) and nicorandil administration were analyzed using Pearson’s correlation coefficient, and multiple regression analysis was performed to identify correlating factors for changes in CAVI. In addition, AHA type B2/C was selected as an objective value that affected change in CAVI after PCI, because it reflects anatomical complexity on coronary arterial lesions and causes procedure-related myocardial injury more frequently. *p* < 0.05 was considered statistically significant. Statistical analysis was performed using JMP computer software version 14.2 (SAS Institute, Cary, NC, USA).

## Results

### Patient background

Baseline patient characteristics are shown in Table 1. Twenty patients were assigned to the control group and 38 patients to the nicorandil group. The proportion of males was higher, and the CAVI tended to be higher in the nicorandil group. Baseline BP was similar between the two groups. In the control group, the number of patients with dyslipidemia and who had a history of heart failure was significantly higher. No significant difference in baseline BNP levels and echocardiographic findings between the groups was found. CO measured by an impedance cardiac output meter tended to be greater in the control group; however, no significant difference was noted. Medication use was similar between the two groups. Details of PCI procedures shown in Table 2. The numbers of target vessels and target lesions that were classified as AHA type B2/C were similar, and drug-eluting stents were used in most patients. Although fluoroscopic time and contrast medium volume tended to be higher in the nicorandil group, no significant difference was observed.

**Table 1:** Baseline patient characteristics

	Control group (*n* = 20)	Nicorandil group (*n* = 38)	*p* value
Male (%)	14 (70.0)	35 (92.1)	0.052
Age (years)	67.6 ± 8.2	68.2 ± 8.6	0.79
Body mass index (kg/m^2^)	24.6 ± 4.5	24.2 ± 2.1	0.72
SBP (mmHg)	138.6 ± 20.5	137.3 ± 19.8	0.81
DBP (mmHg)	82.2 ± 9.2	83.8 ± 9.8	0.55
MBP (mmHg)	101.0 ± 11.4	101.6 ± 12.2	0.84
HR (bpm)	65.6 ± 10.2	62.9 ± 10.8	0.38
CAVI	9.33 ± 1.3	9.76 ± 1.2	0.22
Hypertension (%)	16 (80.0)	26 (68.4)	0.35
Diabetes mellitus (%)	12 (60.0)	21 (55.3)	0.73
Dyslipidemia (%)	19 (95.0)	26 (68.4)	0.023
Current smoker (%)	7 (35.0)	9 (23.6)	0.36
Previous HF (%)	0 (0.0)	8 (21.1)	0.041
Previous MI (%)	8 (40.0)	9 (23.6)	0.19
Previous PCI (%)	14 (70.0)	21 (55.3)	0.28
Previous CABG (%)	3 (15.0)	2 (5.3)	0.33
Previous stroke (%)	1 (5.0)	6 (15.8)	0.40
Number of disease vessel	2.40 ± 0.82	2.11 ± 0.73	0.17
BNP (ng/ml)	50.3 ± 41.0	78.7 ± 122.8	0.56
LVEF (%)	62.8 ± 8.3	62.7 ± 14.0	0.49
*E*/*e*′	12.2 ± 3.3	13.8 ± 7.4	0.76
SV (ml/t)	68.2 ± 17.0	60.5 ± 9.0	0.11
CO (l/min)	4.43 ± 1.4	3.79 ± 0.74	0.069
CI (l/min/m^2^)	2.63 ± 0.74	2.22 ± 0.46	0.064
Estimated SVR (dyne/s/cm^5^)	1886.4 ± 816.0	2049.2 ± 471.1	0.36
Estimated SVRI (dyne/s/cm^5/^m^2^)	3096.7 ± 1088.1	3492.9 ± 785.2	0.52
Medications			
Aspirin (%)	20 (100)	38 (100)	> 0.99
Clopidogrel/prasugrel (%)	20 (100)	37 (97.4)	> 0.99
ACE-I/ARB (%)	11 (55.0)	25 (65.8)	0.42
CCB (%)	16 (80.0)	21 (55.3)	0.062
Beta blocker (%)	9 (45.0)	15 (3.9)	0.68
Diuretics (%)	1 (5.0)	8 (21.1)	0.14
Nitrate (%)	2 (10.0)	9 (23.6)	0.30
Nicorandil (%)	10 (50.0)	13 (34.2)	0.24
Statin (%)	15 (75.0)	26 (68.4)	0.60
Hypoglycemic agent	7 (35.0)	10 (26.3)	0.50
Insulin therapy	0 (0.0)	4 (10.5)	0.29

Data are presented as means ± standard deviation or number of subjects (%)

*SBP* systolic blood pressure, *DBP* diastolic blood pressure, *MBP* mean blood pressure, *HR* heart rate, *CAVI* cardio-ankle vascular index, *HF* heart failure, *MI* myocardial infarction, *PCI* percutaneous coronary intervention, *CABG* coronary artery bypass grafting, *BNP* brain natriuretic peptide, *LVEF* left ventricular ejection fraction, *SV* stroke volume, *CO* cardiac output, *CI* cardiac index, *SVR* systemic vascular resistance, *SVRI* systemic vascular resistance index, *ACE-I* angiotensin-converting enzyme inhibitor, *ARB* angiotensin II receptor blocker, *CCB* Ca^2+^ channel blocker

**Table 2:** Details of PCI

	Control group (*n* = 20)	Nicorandil group (*n* = 38)	*p* value
Target vessel			
RCA (%)	6 (30.0)	10 (26.3)	0.77
LMT (%)	1 (5.0)	3 (7.9)	> 0.99
LAD (%)	11 (55.0)	18 (47.4)	0.58
LCX (%)	3 (15.0)	13 (34.2)	0.11
AHA type B2/C (%)	11 (55.0)	24 (63.2)	0.55
DES (%)	18 (90.0)	36 (94.7)	0.6
BMS (%)	0 (0.0)	2 (5.3)	0.54
POBA (%)	2 (10.0)	0 (0.0)	0.11
Fluoroscopic time (min)	22.9 ± 14.7	29.2 ± 22.4	0.26
Contrast medium (ml)	110.4 ± 24.0	129.0 ± 44.9	0.073

Data are presented as means ± standard deviation or number of subjects (%)

*PCI* percutaneous coronary intervention, *RCA* right coronary artery, *LMT* left main trunk, *LAD* left anterior descending artery, *LCX* left circumflex artery, *AHA* American Heart Association, *DES* drug-eluting stent, *BMS* bare metal stent, *POBA* plain old balloon angioplasty

### Changes in troponin I and BNP immediately after PCI and at 12 h post-PCI

**Fig. 1:**
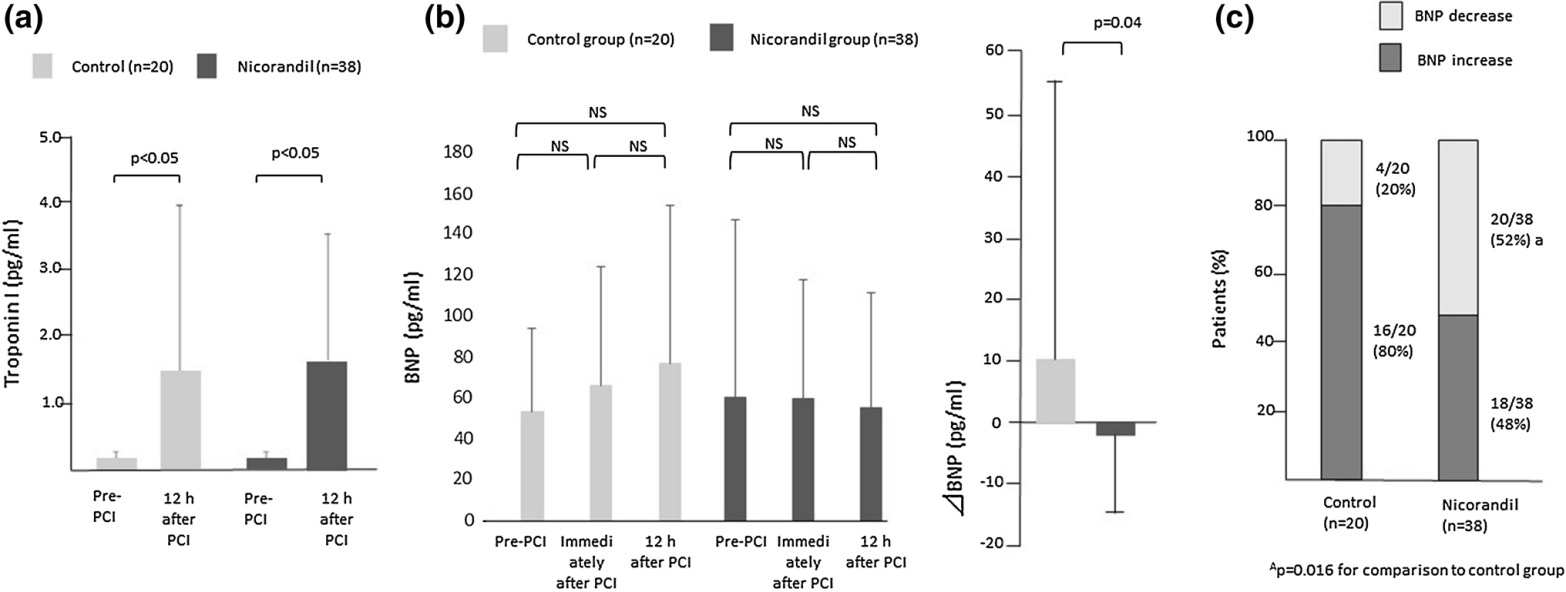
**a** Changes in troponin I with or without nicorandil administration after PCI. **b** Changes in BNP and ∆BNP with or without nicorandil administration. **c** Percentages of patients with decreased or increased brain natriuretic peptide after PCI. Data are presented as means ± standard deviation or number of subjects (%). *BNP* brain natriuretic peptide, *PCI* percutaneous coronary intervention

Troponin I values before and 12 h after PCI in both groups are shown in Fig. 1a. Troponin I was significantly elevated in both groups regardless of nicorandil administration. Changes in BNP before and after PCI and after nicorandil administration are shown in Fig. 1b. No significant differences in BNP value before, immediately after, and 12 h after PCI in both groups were found (control group: 50.3 ± 41.0 pg/ml [pre], 62.3 ± 58.1 pg/ml [after PCI], and 72.5 ± 77.2 pg/ml [12 h after PCI] vs. nicorandil group: 78.7 ± 122.8 pg/ml [pre], 77.3 ± 98.7 pg/ml [after PCI], and 70.9 ± 86.3 pg/ml [12 h after PCI)]). Changes in BNP between immediately after PCI and 12 h post-PCI were significantly different between the two groups (10.8 ± 44.2 pg/ml in the control group and − 2.6 ± 14.6 pg/ml in the nicorandil group; *p* = 0.04). The percentages of patients with increased and decreased BNP in both groups are shown in Fig. 1c. In the control group, BNP increased in 80% of the patients 12 h after PCI. In the nicorandil group, BNP increased in 48% of the patients and decreased in 52% after nicorandil administration. The percentage of patients with decreased BNP was significantly higher in the nicorandil group (20% vs. 52%; *p* = 0.016).

### Changes in vascular parameters immediately and at 12 h after PCI

**Fig. 2:**
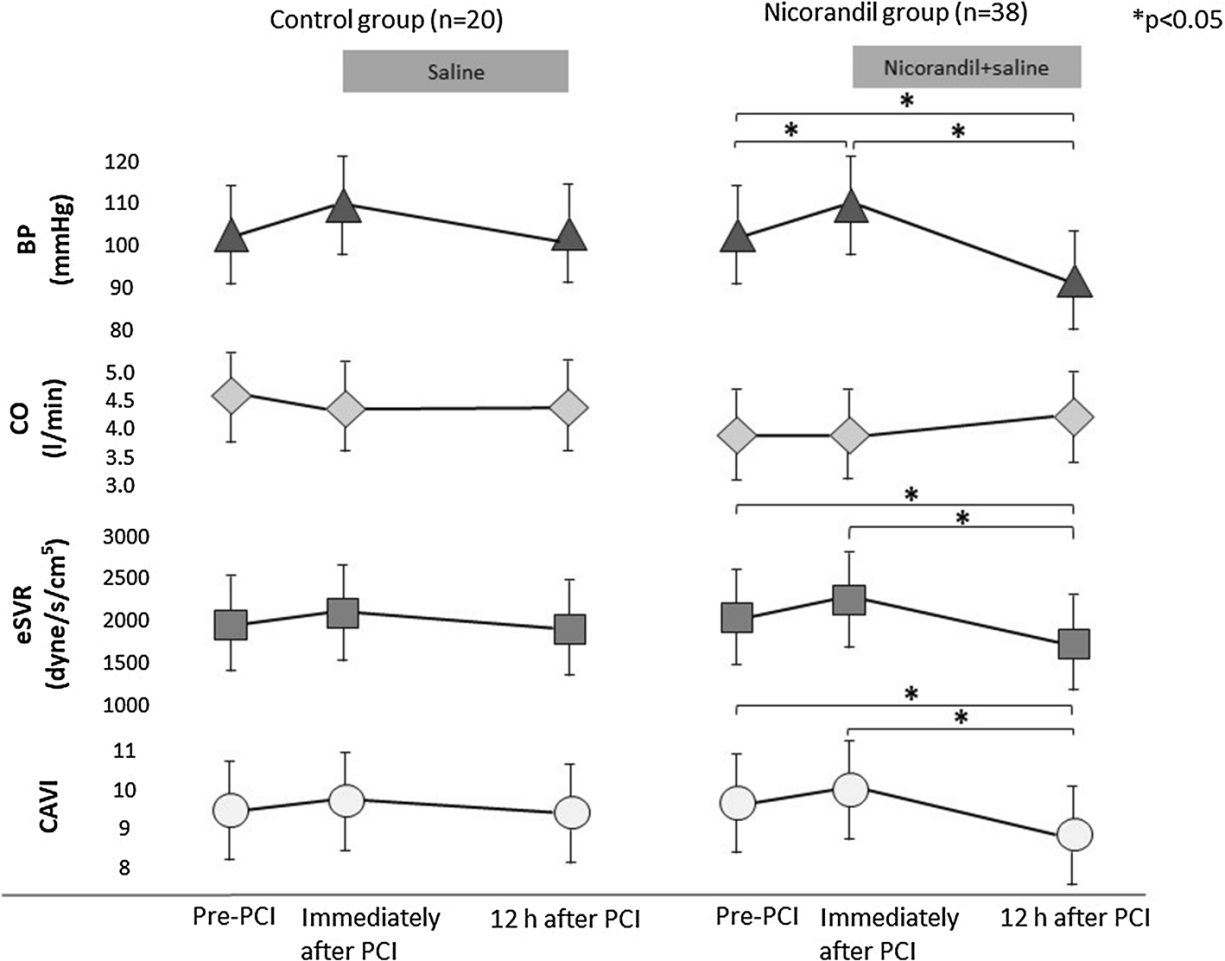
Changes in CAVI and various circulation parameters with or without nicorandil administration. Data are presented as means ± standard deviation. *CAVI* cardio-ankle vascular index, *BP* blood pressure, *CO* cardiac output, *eSVR* estimated systemic vascular resistance, *PCI* percutaneous coronary intervention

Changes in vascular parameters immediately and at 12 h after PCI in both groups are shown in Fig. 2. Immediately after PCI, the BP, eSVR, and CAVI increased; however, no significant differences in these parameters, except BP in the nicorandil group, were observed. At 12 h after PCI, BP, eSVR, and CAVI slightly decreased compared to those immediately after PCI in the control group; in the nicorandil group, the parameters decreased significantly (BP 108.8 ± 11.1–88.1 ± 9.9 mmHg; eSVR, 2286.8 ± 697.0–1678 ± 547.6 dyne/s/cm^5^/m^2^; CAVI, 10.1 ± 1.1–8.8 ± 1.3; *p* < 0.0001). CO tended to increase in the nicorandil group; however, no significant difference was noted.

### Factors for change in CAVI after PCI

Factors correlated with the change in CAVI after coronary intervention are shown in Table 3. In single linear analysis; AHA type B2/C (*r* = − 0.27, *p* = 0.04), change in BP (*r* = 0.36, *p* < 0.01) and nicorandil administration (*r* = − 0.47, *p* < 0.001) were significantly correlated with change in CAVI. Change in CO tended to have a negative correlation, while a change in eSVR tended to have a positive correlation with the change in CAVI, although not statistically significant. Multiple regression analysis revealed that change in CO, change in eSVR, and nicorandil administration were independent contributing factors for change in CAVI after PCI.

**Table 3:** Factors correlated with change in CAVI after coronary intervention in all patients

	Single linear analysis		Multiple regression analysis		
	*R*	*p* value	SE	*t* value	*p* value
AHA type B2/C	− 0.27	0.04	0.22	− 1.9	0.07
∆BP	0.36	< 0.01	0.02	1.7	0.09
∆CO	− 0.14	0.29	0.21	− 2.2	0.03
∆eSVR	0.14	0.29	0.0004	− 2.2	0.03
Nicorandil administration	− 0.47	< 0.0001	0.31	− 2.5	0.01

*R*^2^ = 0.34, *F* value 5.4, *p* < 0.001

*R* Pearson’s correlation coefficient, *SE* standard error, *CAVI* cardio-ankle vascular index, *AHA* American Heart Association, *BP* blood pressure, *CO* cardiac output, *eSVR* estimated systemic vascular resistance

## Discussion

In this study, patients with stable angina who underwent PCI received nicorandil after PCI, and their cardiac burden and CAVI were compared with those of the patients who did not receive nicorandil. Serum troponin I levels increased at 12 h after PCI in the control and nicorandil groups, indicating that PCI has an injurious effect on the myocardium. ΔBNP between immediately after PCI and 12 h post-PCI was significantly higher in the control group than in the nicorandil group, which suggested that nicorandil administration after PCI may have some preventive effect on the myocardium and cardiac function. While BNP level improved in the nicorandil group at 12 h after PCI, no improvement in troponin I was noted, which could be attributed to a delayed turnover rate of troponin I in the blood.

To identify the mechanism of how nicorandil administration improved cardiac function, various vascular parameters were analyzed. BP, eSVR, and CAVI increased simultaneously immediately after PCI, although not statistically significant. This finding indicates that PCI may induce stress, especially on the arteries and the myocardium. The stress may enhance the contraction of the systemic arterial wall, which could be explained by sympathetic nerve activation, resultantly increasing BP, eSVR, and CAVI. Enhanced arterial stiffness shown by the increment of CAVI is thought to result in high afterload on the left ventricle, similar to the increment of BP and vessel resistance, which can deteriorate cardiac function. BP, eSVR, and CAVI at 12 h after PCI tended to slightly decrease in the control group, whereas significantly decreased in the nicorandil group compared with those immediately after PCI. In addition, improvement of BNP between immediately after PCI and 12 h after PCI was significantly greater in the nicorandil group. The mechanism of improvement BNP may be related to the results of lowering peripheral vascular resistance and arterial stiffness shown by CAVI (Fig. 2). These results indicated that nicorandil improved not only coronary circulation, including microcirculation by dilating coronary resistance arteries, but also improved systemic circulation by dilating systemic peripheral arteries. Resultantly, these effects by nicorandil could relief afterload on the left ventricle and improve cardiac function, thereby reducing the cardiac burden and BNP level. Moreover, CAVI value after nicorandil administration decreased to 8.8 ± 1.3, which was lower than that of pre-PCI. This result suggests that arterial stiffness may be enhanced through activation of sympathetic nerve in patients with coronary artery disease due to anxiety of being hospitalized and/or receiving PCI in pre-PCI, and the improvement of the enhanced arterial stiffness may be affected by the improvement of functional stiffness due to smooth muscle cell relaxation. Therefore, these conditions before PCI might have also led to the BNP decrease. Enhanced arterial stiffness may be involved in hemodynamic conditions as enhanced afterload on the left ventricle, and nicorandil may be useful to reduce the enhancement afterload. Additionally, there are several reports that nicorandil could relieve the sympathetic nerve activation in heart failure patients [23, 24]. In addition to afterload reduction due to the vasodilating effect, nicorandil improves sympathetic nerve activation, which may also lead to a reduction in the cardiac burden after PCI in this study subjects.

This study is the first report to quantify arterial stiffness after PCI using CAVI in patients with stable angina and to examine the relationship of CAVI with circulation parameters. Factors that correlated with the change in CAVI after PCI were the change in BP and nicorandil administration in a single linear analysis. Whereas, in multiple regression analysis, changes in CO and eSVR were selected as independent contributing factors for change of CAVI in addition to nicorandil administration (Table 3). These results suggested that the predominant factor of improvement of CAVI may be decrement of vessel resistance and increment of CO independent from BP along with nicorandil administration. Nicorandil may improve afterload, including arterial stiffness, resultantly decreased BP. Several studies on the change in arterial stiffness based on CAVI during the administration of vasoactive drugs, such as α-blocker, nitroglycerin, prostaglandin I2, and Ca^2+^ antagonist, in the acute phase have been reported [19–22]. In some of these studies, CAVI reacted differently with BP and vessel resistance in the acute phase of drug administration. These findings indicate that arterial stiffness evaluated by CAVI may play an important role in the maintenance of hemodynamics different from BP and vessel resistance. Further studies are needed to clarify the meaning of CAVI measurement, but CAVI may be useful to evaluate arterial stiffness as an afterload indicator on systemic circulation. In this study, nicorandil administration after PCI improved cardiac burden accompanied by improved arterial stiffness monitored with CAVI. However, to confirm the effect of nicorandil administration on the cardiac burden in patients receiving PCI and to clarify the precise mechanism, further studies, including an investigation on nicorandil administration in the acute phase, are warranted.

This study has some limitations. First, this study is an observational study with a small number of patients. Second, as this study involves patients with stable angina, the findings are not applicable to those with acute myocardial infarction. Lastly, evaluation of preload was not performed, and the interaction between nicorandil and conventional medicine was not investigated.

## Conclusions

PCI could result in myocardial injury and/or cardiac burden, and nicorandil administration after PCI may be effective in partly relieving the burden by decreasing arterial stiffness.
